# Effect of dietary seaweed (*Ascophyllum nodosum*) supplementation on milk mineral concentrations, transfer efficiency, and hematological parameters in lactating Holstein cows

**DOI:** 10.3168/jds.2022-23074

**Published:** 2023-10

**Authors:** E.E. Newton, K. Theodoridou, M. Terré, S. Huws, P. Ray, C.K. Reynolds, N. Prat, D. Sabrià, S. Stergiadis

**Affiliations:** 1 School of Agriculture, Policy and Development, University of Reading, Reading, RG6 6EU, United Kingdom; 2 Queen's University Belfast, Institute for Global Food Security, Belfast, BT9 5DL, United Kingdom; 3 Department of Ruminant Production, Institute de Recerca i Tecnologia Agroalimentàries, Caldes de Montbui, 08140, Spain; 4 The Nature Conservancy, Arlington, VA 22203

**Keywords:** bovine milk, minerals, seaweed, iodine, *Ascophyllum nodosum*

## Abstract

This study investigated the effect of feeding seaweed (*Ascophyllum nodosum*) to dairy cows on milk mineral concentrations, feed-to-milk mineral transfer efficiencies, and hematological parameters. Lactating Holstein cows (n = 46) were allocated to 1 of 2 diets (n = 23 each): (1) control (CON; without seaweed) and (2) seaweed (SWD; replacing 330 g/d of dried corn meal in CON with 330 g/d dried *A. nodosum*). All cows were fed the CON diet for 4 wk before the experiment (adaptation period), and animals were then fed the experimental diets for 9 wk. Samples included sequential 3-wk composite feed samples, a composite milk sample on the last day of each week, and a blood sample at the end of the study. Data were statistically analyzed using a linear mixed effects model with diet, week, and their interaction as fixed factors; cow (nested within diet) as a random factor; and data collected on the last day of the adaptation period as covariates. Feeding SWD increased milk concentrations of Mg (+6.6 mg/kg), P (+56 mg/kg), and I (+1,720 μg/kg). It also reduced transfer efficiency of Ca, Mg, P, K, Mn, and Zn, and increased transfer efficiency of Mo. Feeding SWD marginally reduced milk protein concentrations, whereas there was no effect of SWD feeding on cows' hematological parameters. Feeding *A. nodosum* increased milk I concentrations, which can be beneficial when feed I concentration is limited or in demographics or populations with increased risk of I deficiency (e.g., female adolescents, pregnant women, nursing mothers). However, care should also be taken when feeding SWD to dairy cows because, in the present study, milk I concentrations were particularly high and could result in I intakes that pose a health risk for children consuming milk.

## INTRODUCTION

Dairy products are significant sources of dietary essential minerals such as Ca, Se, Mg, Zn, and I across the world for multiple demographics, and the extent of their contribution is influenced by country-specific agricultural and dietary circumstances (Haug et al., 2007; Hettinga and van Valenberg, 2017; Smith et al., 2022). Macrominerals Ca and Mg are necessary for the development and maintenance of healthy bones (Thorning et al., 2016). Microminerals Se and Zn play an important role in numerous biochemical pathways and cellular functions in human bodies (Chasapis et al., 2020; Kieliszek et al., 2022). Finally, sufficiency of I is important not only for the avoidance of goiter, but also for fetal and infant neurological development, as even mild maternal deficiency has been found to negatively affect intelligence quotients of their offspring (Bath et al., 2013).

Mineral concentrations in bovine milk can be modulated by a host of factors such as breed, feed composition, stage of lactation, climate, and processing, of which one of the most influential is seasonal differences (Nada et al., 2010; Stergiadis et al., 2021). Given that climate change continues to pose a substantial barrier to the attainment of food security at the farm level, production systems must adapt quickly to maintain production and product quality while adopting potentially sweeping changes (Gornall et al., 2010; Thornton et al., 2018; Ahmed et al., 2022). In turn, changes to feeding regimens with differing mineral concentrations may affect their concentrations in milk and dairy products (Newton et al., 2021; Stergiadis et al., 2021). The term “mineral transfer efficiency” has been used to describe the proportions of elements consumed that are secreted in milk (Kronqvist, 2011; Newton et al., 2021). Transfer efficiencies are a function of dietary supply and can be affected by the mineral content of the feed and the interaction that certain feed components have on minerals' bioavailability and absorption, such as glucosinolates that are found in commonly fed cruciferous vegetables and are known to reduce I transfer from feed to milk (Papas et al., 1979; Bischoff, 2016). Therefore, any systemic changes to feeding practice on the farm, specifically regarding diet composition, should be contextually assessed to gauge the transfer efficiency of minerals to milk and the resulting impacts on nutritional security of dairy consumers.

The inclusion of aquacultural products into dairy cattle feed, specifically that of seaweed or seaweed-based products (such as certain species of red seaweed within the *Asparagopsis* genus, or certain species of brown seaweed within the *Ascophyllum* genus), has recently gained interest as a way of reducing enteric methane emission and promoting small-holder resiliency (Duarte et al., 2017). Recent research has shown marked reduction of methane emissions in ruminants, when *Asparagopsis armata* and *Asparagopsis taxiformis* were offered in the diets of dairy cows (−43%) and beef steers (−80%), respectively (Roque et al., 2019, 2021). Additionally, brown seaweed *Ascophyllum nodosum* feeding has been shown to increase I content of milk by +1,192 μg/kg when fed at 170 g/cow per d, in comparison with a control diet (Antaya et al., 2015, 2019). Similar results have also been observed when a 91:9 mixture of brown seaweeds *A. nodosum:Laminaria digitata* was fed at 158 g/cow per d, leading to an increase of +1,649 μg/kg milk when compared with the control diet (Newton et al., 2021). Seaweeds can be a rich source of minerals due to their large capacity for mineral bioaccumulation, but their concentrations can vary across species, seasons, and processing methods among other factors (Nitschke and Stengel, 2015, 2016). Alongside the potential accumulation of beneficial minerals, there are concerns that some harvested seaweeds (either wild or cultivated) may contain heavy metals that are potentially harmful to animals physiologically and subsequently to the consumer of animal-based products (Roleda et al., 2018).

The brown seaweed *A. nodosum* represents a significant portion of current research interest, specifically in Europe, wherein it is by far the species with the largest wild collection for Ireland and Iceland, as well as placing fourth in highest tonnage collected in the world (FAO, 2021). Therefore, *A. nodosum* is a rather plentiful and accessible seaweed for feeding to livestock in Europe. However, if seaweed is to be used as a widespread feed ingredient for dairy cows, effects and persistency of effects on milk composition must be considered. Therefore, the present study aimed to (1) investigate the effect of feeding 330 g/d dried *A. nodosum* to Holstein dairy cows on DMI, milk yield and composition, including concentrations of milk macrominerals and trace elements, (2) quantify the effect on animal hematological parameters, and (3) estimate the impact that the consumption of milk from seaweed-fed cows may have on consumer mineral intakes.

## MATERIALS AND METHODS

### Experimental Conditions

The study was conducted at Institute of Agrifood Research and Technology (IRTA) dairy research farm in Monells (Girona, Spain) from June to August 2021. In total, 23 primiparous and 23 multiparous Holstein dairy cows were selected based on initial BW ± standard deviation (697 ± 65.6 kg), average milk yield (33.9 ± 5.27 kg/d), and DIM (168 ± 59.8). Cows were blocked by parity (primiparous and multiparous), DIM, and milk yield, and randomly assigned to 2 dietary treatments equally distributed in 4 different pens, within a randomized complete block design in which they remained in the experimental diets and groups across the 9-wk experiment. All cows (from both groups) were fed the CON diets continuously for 4 wk before the introduction to the experimental diets (adaptation period). Diets were fed as a TMR at 48:52 forage:concentrate ratio and represented a control diet (without seaweed; **CON**) and a diet replacing 330 g/d of dried corn meal with 330 g/d of dried seaweed (*A. nodosum*; **SWD**; SeaLac Ltd., Kiltimagh, Ireland). Animals were fed twice daily at 0800 and 1900 h for a period of 64 d, and all received a total of 300 g/d dried pelleted soybean in the milking parlor (150 g of dried soy per milking). Pens were equipped with 20 cubicles bedded with a mixture of compost and sawdust, 4 electronic water troughs, and 15 electronic feed bins (MooFeeder, MooSystems, Cortes, Spain) that allowed the registration of individual daily feed intake by identifying the animal when it entered into the feed bin and by the difference between the weight of feed before and after feeding. Diet formulations are presented in Table 1. Mineral concentrations of TMR fed to cows of the experimental diets are presented in Table 2.

**Table 1:** Ingredients and basic composition of TMR of cows fed the experimental diets (control, no seaweed, CON; 330 g/d dried *Ascophyllum nodosum* supplement, SWD)

Item	CON	SWD
Diet formulation (% of DM)		
Corn meal	19.8	18.7
Alfalfa silage	15.9	15.9
Ryegrass silage	13.2	13.2
Soybean hulls	10.2	10.2
Corn silage	9.3	9.4
Oat hay	9.0	9.0
Wheat meal	8.8	8.8
Soybean meal	8.2	8.2
Wheat middlings	3.8	3.8
Barley straw	0.8	0.8
Calcium carbonate	0.4	0.3
Magnesium oxide	0.2	0.2
Premix^1^, ^2^	0.2	0.2
Sodium chloride	0.2	0.1
*A. nodosum*	0.0	1.2
TMR basic composition (%)		
NDF	42.6	41.9
ADF	27.8	26.1
CP	16.8	16.3
Ash	9.2	9.0
Fat	3.2	3.1

1 Premix contained: vitamin A: 2,250,000 IU/kg; vitamin D_3_: 665,000 IU/kg; vitamin E: 8,800 mg/kg; manganese (manganese oxide): 30,000 mg/kg; copper (copper sulfate): 5,000 mg/kg; zinc (zinc oxide): 30,000 mg/kg; iodine (potassium iodide): 250 mg/kg; cobalt (cobalt acetate): 40 mg/kg; selenium (sodium selenite): 150 mg/kg; iron (iron carbonate): 20,000 mg/kg, butylhydroxytoluene: 1,500 mg/kg, sepiolite: 279,949 mg/kg.

2 Iodine was removed from the premix in the SWD ration to ensure that I supply in the diet does not exceed EFSA's regulations of 5 mg of I/kg of DM (EFSA, 2013).

**Table 2:** Mineral concentrations of TMR fed to cows of the experimental diets (control, no seaweed, CON; 330 g/d dried *Ascophyllum nodosum* supplement, SWD) and cows' total mineral intake per day^1^

Item	CON (n = 9)			SWD (n = 9)		
	AVG	SD	Range	AVG	SD	Range
Macromineral concentration of experimental diet^2^ (g/kg DM)						
Calcium	6.84	0.311	6.54–7.34	7.04	0.212	6.78–7.36
Magnesium	2.58	0.047	2.52–2.65	2.84	0.154	2.62–3.01
Phosphorus	3.96	0.121	3.75–4.06	4.15	0.067	4.08–4.24
Potassium	19.38	1.070	18.21–21.03	19.49	0.829	18.62–20.33
Sodium	1.44	0.019	1.41–1.46	1.17	0.055	1.10–1.25
Trace element concentration of experimental diet^2^ (mg/kg DM unless indicated by^3^)						
Copper	17.15	1.407	15.11–18.91	13.00	1.962	10.91–16.20
Iron	211	42.9	140–251	634	45.3	605–712
Iodine^3^	509	77.7	424–635	6,087	1,534.8	4,217–8,470
Manganese	35.2	3.71	30.2–40.1	86.8	8.58	75.2–97.7
Molybdenum	1.39	0.030	1.35–1.43	1.14	0.021	1.11–1.16
Zinc	33.4	4.23	26.4–36.7	70.6	5.98	62.6–77.4
Heavy metal concentration of experimental diet^2^ (mg/kg DM unless indicated by^3^)						
Arsenic^3^	195	10.1	181–205	452	38.1	396–496
Cobalt	389	121.8	307–598	329	120.3	225–533
Macromineral intake from experimental diet^4^ (g/d)						
Calcium	165	21.5	120–220	179	22.7	137–226
Magnesium	62.1	6.65	46.6–77.2	71.9	9.33	53.6–92.3
Phosphorus	95.3	11.91	67.8–121.7	105.3	13.07	79.0–130.0
Potassium	467	63.6	338–630	495	63.9	374–621
Sodium	34.5	3.92	25.8–43.8	29.7	3.83	22.8–38.0
Trace element intake from experimental diet^4^ (mg/d unless indicated by^5^)						
Copper	414	66.9	273–566	331	71.8	219–497
Iron^5^	5.08	1.181	2.92–7.53	16.10	2.446	11.74–21.83
Iodine	12	2.30	8.87–18.5	153	37.9	88–238
Manganese	843	109.7	631–1,105	2,203	351.1	1,456–2,997
Molybdenum	33.4	3.48	25.8–41.9	28.9	3.62	22.2–35.5
Zinc	801	127.1	552–1,098	1,790	263.8	1,214–2,376
Heavy metal intake from experimental diet^4^ (mg/d)						
Arsenic	4.68	0.551	3.67–6.15	11.44	1.541	8.30–14.9
Cobalt	9.38	3.918	6.06–17.41	8.40	3.372	4.52–16.26

1 AVG = average value; range = minimum and maximum values.

2 Concentrations of minerals in experimental diets account for all wk 2 to 9 of the 9-wk experiment.

3 Expressed in micrograms per kilogram DM.

4 Concentrations of mineral intakes in experimental diets account for wk 2, 4, 6, and 8 of the 9-wk experiment.

5 Expressed in grams per day DM.

### Experimental Sampling

Samples of TMR were obtained weekly to determine DM and mineral concentrations. Samples of feed were frozen at −20°C and composited every 3 wk and analyzed. Fortnightly, individual milk samples were collected for morning and afternoon milking and composited on the basis of milk yield to produce a daily sample for each cow. Two EDTA-treated tubes (Vacutainer, Becton Dickinson, Madrid, Spain) for blood samples (5 mL) were obtained from the coccygeal vein at d 64 of the study. One tube was refrigerated for further hematological analysis, and the other was centrifuged at 1,500 × *g* for 10 min at 5°C and the resulting plasma frozen at −20°C.

### Experimental Analysis

Feed was analyzed for DM (method 934.01; oven-drying in 100°C until constant weight), N (method 984.13; copper catalyst Kjeldahl method), ether extract (method 920.39; ether extraction), and ash (method 942.05; heat at 600°C for 2 h) following AOAC (1990) and NDF according to Van Soest et al. (1991) using sodium sulfite and heat-stable amylase and expressed inclusive of residual ash. Nonfiber carbohydrates were calculated as 100 minus the sum of CP, NDF, ether extract, and ash. Milk was analyzed for fat, protein, lactose, and urea concentrations using infrared spectroscopy (MilkoScan 7; Foss Iberia S.A., Barcelona, Spain) and SCC were analyzed by Fossomatic 7 (Foss Iberia S.A.).

Mineral concentrations of feed and milk were determined by utilizing a protocol based on US-EPA method 3051A (microwave-assisted acid digestion of sediment, sludges, soils, and oils; EPA, 2007), using inductively coupled plasma mass spectrometry (Agilent 7000, Agilent, Singapore). Modifications included the amount of milk and acid that was used to digest the milk, as well as the concentration of diluted solution that was then analyzed, described below. All samples analyzed for mineral concentrations were assessed in the present study in duplicates and were validated using ERM-BD150 certified reference material (CRM) skim milk powder for milk, and IPE 993 black poplar hybrids leaf (*Populus* × *euramericana*) from Lienden, the Netherlands, for TMR. Seaweed I concentrations were validated in reference to a chemistry analysis report generated by JHG Analytical Services Ltd. in place of a specific *A. nodosum* reference material. Digestion and subsequent extraction was accomplished using Ethos Easy Microwave Digestion System with the heating of a 7.5 mL of HNO_3_ + 2.5 mL of HCl solution, and either 1 mL of milk or 0.5 g of feed to form a solution. The subsequent acid and sample solution was then subjected to a 15 min heating phase to reach 180°C, maintained at 180°C for 10 min, and then allowed to cool until it reached ambient temperature. The digested solution from microwave vessels was filtered through Cytiva Whatman 540 hardened ashless 110-mm diameter filter paper into Corning Falcon 50-mL polypropylene conical centrifuge tubes. The resulting solution was then diluted to a total weight of 50 g with ultrapure H_2_O, and then again at factors of 1:4 and 1:10 with ultrapure H_2_O into Corning Falcon 15-mL polypropylene conical centrifuge tubes for analysis.

Standards were created and later adjusted to encompass expected sample values based on preliminary testing within the same acid concentration for each final diluted sample. Trace element (Mn, Fe, Co, Cu, Zn, As, Mo) standards except for I were created with SPEX CertiPrep multi-element standard. Standards for I were created with ROMIL PrimAg Mono-Component Reference Solutions. Macromineral (Ca, Mg, P, K, Na) standards were created with the element specific Fisher Chemical 1,000 ppm standard.

Mineral concentrations (mg/kg milk) were calculated as follows:

$$\frac{\left[analytical\;reading\;\left(\frac{\mu g}{L}\right)-blank\;\left(\frac{\mu g}{L}\right)\right]\times dilutionfactor}{1,000\;\times \left(\frac{measured\;sample\;mass\left(g\right)}{diluted\;solution\;mass\left(g\right)}\right)},$$

where (1) dilution factor was the fractional dilution performed to maintain read values within standard ranges (4 and 10 for micro- and macrominerals, respectively), (2) diluted solution mass was the total mass of the solution when brought up to 50 g with ultrapure H_2_O, and (3) measured sample mass was the weight of the sample delivered to the microwave digestion vessel.

Transfer efficiencies from feed to milk (g into milk per 100 g ingested) were calculated as follows:

$$[\begin{array}{l} 100\;\times \\ \left\{\frac{\left[milk\;mineral\;concentration\left(\frac{\mu g}{kg\;milk}\right)\times milk\;output\;\left(\frac{kg\;}{d}\right)\right]}{\left[diet\;mineral\;concentration\left(\frac{\mu g}{kg\;DM}\right)\times feed\;intake\left(\frac{kg\;DM}{d}\right)\;\right]}\right\}. \end{array}$$

Plasma haptoglobin was measured using the commercial kit Tridelta PHASE haptoglobin assay (Tridelta Development Ltd., Maynooth, Ireland). Whole blood in EDTA tubes was refrigerated for analysis in the following 12 h for hematological parameters (white blood cell count, neutrophils, lymphocytes, monocytes, and eosinophils) using an Element HT5 analyzer (Heska, Loveland, CO).

### Statistical Analysis

Data for milk production and composition, efficiency parameters, and mineral concentrations and transfer efficiencies were analyzed using a linear mixed effects model in Minitab 20 (Minitab LLC, State College, PA). Diet, week, and their interaction were used as fixed factors, and cow (nested within treatment) as a random factor. Data collected on the last day of the adaptation period (before introducing experimental diets) were used as covariate for all measured variables, except for mineral transfer efficiencies. Normality of residuals were evaluated visually, and no data showed deviation from normality except for SCC, which was log-transformed before performing the linear mixed model. Where necessary, Tukey's least significant difference test (*P* < 0.05) was used for pairwise comparison for the means, where the mixed effects model showed a significant effect of week or the diet × week interaction. Hematological data were analyzed by general linear models in Minitab 20, using diet as fixed factor.

## RESULTS

### Milk Basic Composition and Efficiency Parameters

**Figure 1:**
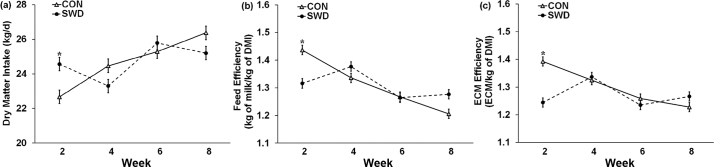
Interaction means ± SE (error bars) for the effects of dietary treatment (control, no seaweed, CON; 330 g/d dried *Ascophyllum nodosum* supplement, SWD) and week (2, 4, 6, and 8) on animal DMI (a; kg/d), milk feed efficiency (b; kg of milk/kg of DMI; *P* < 0.001), and ECM efficiency (c; ECM/kg of DMI; *P* < 0.001). Means for diet treatments within a week denoted with * are significantly different (*P* < 0.05).

There was a significant effect of diet on the concentrations of protein (*P* = 0.016) in milk (Table 3), but the numerical differences were small with SWD milk containing 0.06 g less protein per 100 g of milk, when compared with CON milk. There was a significant effect (*P* < 0.001) of the diet × week interaction for DMI, feed, and ECM efficiency (Table 3). In wk 2, SWD-fed cows has higher DMI but lower efficiency measurements than CON cows; but these differences were not significant in the following weeks (Figure 1).

**Table 3:** Means, SE, and ANOVA *P*-values for the effect of dietary treatment (control, no seaweed, CON; 330 g/d *Ascophyllum nodosum* supplement, SWD) on animal diet data, milk production and basic composition, and efficiency parameters

Parameter	Dietary treatment^1^			ANOVA *P*-value^2^		
	CON (n = 92)	SWD (n = 92)	SE	Diet	Week	Diet × week
DMI (kg/d)	24.7	24.7	0.34	0.972	<0.001	<0.001
Milk yield (kg/d)	32.1	32.2	0.29	0.806	0.480	0.398
Milk fat (g/100 g milk)	3.63	3.65	0.058	0.841	<0.001	0.563
Milk protein (g/100 g milk)	3.38	3.32	0.016	0.016	<0.001	0.460
Milk lactose (g/100 g milk)	4.91	4.89	0.014	0.293	<0.001	0.124
Milk urea (mg/L)	225	212	7.2	0.120	<0.001	0.470
Milk SSC^3^ (× 1,000/mL)	143	85	36.8	0.677	0.025	0.973
Milk fat:protein	1.07	1.10	0.015	0.128	0.013	0.557
ECM^4^	31.7	31.6	0.34	0.918	0.001	0.328
Feed efficiency (kg milk/kg of DMI)	1.31	1.31	0.01	0.865	<0.001	<0.001
ECM efficiency (ECM/kg of DMI)	1.30	1.27	0.011	0.072	<0.001	<0.001

1 n = number of records.

2 Significances were declared at *P* < 0.05.

3 *P*-values were generated from the common logarithm of SCC values.

4 Energy-corrected milk yield = milk yield (kg) × [0.01 + 0.0122 milk fat (g/kg) + 0.0077 milk protein (g/kg) + 0.053 milk lactose (g/kg)].

### Milk Mineral Composition and Transfer Efficiencies

**Figure 2:**
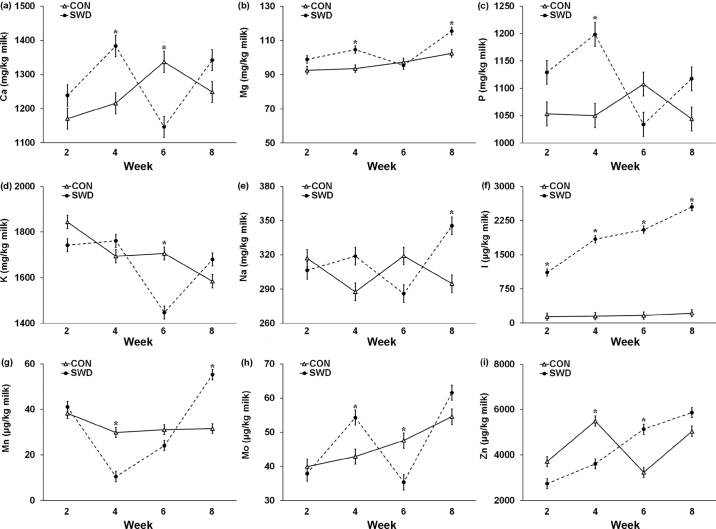
Interaction means ± SE (error bars) for the effects of dietary treatment (control, no seaweed, CON; 330 g/d dried *Ascophyllum nodosum* supplement, SWD) and week (2, 4, 6, and 8) on the concentration of Ca (a; mg/kg; *P* < 0.001), Mg (b; mg/kg; *P* = 0.001), P (c; mg/kg; *P* < 0.001), K (d; mg/kg; *P* < 0.001), Na (e; mg/kg; *P* < 0.001), I (f; μg/kg; *P* < 0.001), Mn (g; μg/kg; *P* < 0.001), Mo (h; μg/kg; *P* < 0.001), and Zn (i; μg/kg; *P* < 0.001) in milk. Means for diet treatments within a week denoted with * are significantly different (*P* < 0.05).

There was a significant effect of diet on the concentrations of Mg (*P* = 0.007), P (*P* = 0.030), and I (*P* < 0.001) in milk (Table 4). The SWD diet, compared with CON diet, increased Mg by 6.8% (+6.6 mg/kg milk), P by 5.3% (+56 mg/kg milk), and I by 1,036% (+1,720 μg/kg milk). There was a significant effect of the diet × week interaction (*P* ≤ 0.001) for all measured mineral concentrations (Ca, Mg, P, K, Na, I, Mn, Mo, and Zn; Figure 2). The SWD milk contained more Ca, P, and Mo than CON milk in wk 4, but not in wk 8. The SWD milk contained more Mg in wk 4 and 8, Zn in wk 6, and Na in wk 8. The SWD milk contained less K in wk 6, Mo in wk 6, Zn in wk 4, Ca in wk 6, as well as Mn in wk 4. Milk I concentrations were higher in SWD than CON milk throughout the experiment with the relative differences incrementally increasing from wk 2 to 8.

**Table 4:** Means, SE, and ANOVA *P*-values for the effect of dietary treatment (control, no seaweed, CON; 330 g/d *Ascophyllum nodosum* supplement, SWD) on mineral concentrations of milk and feed-to-milk transfer efficiency

Mineral	Dietary treatment^1^			ANOVA *P*-value^2^		
	CON (n = 92)	SWD (n = 92)	SE	Diet	Week	Diet × week
Macromineral concentration (mg/kg)						
Calcium	1,243	1,279	18.0	0.183	0.003	<0.001
Magnesium	96.8	103.4	1.63	0.007	<0.001	<0.001
Phosphorus	1,064	1,120	17.5	0.030	0.004	<0.001
Potassium	1,707	1,658	21.0	0.088	<0.001	<0.001
Sodium	305	314	5.7	0.262	0.010	<0.001
Trace element concentration (μg/kg)						
Iodine	166	1,886	70.1	<0.001	<0.001	<0.001
Manganese	32.7	32.8	1.45	0.970	<0.001	<0.001
Molybdenum	46.2	47.3	1.60	0.657	<0.001	<0.001
Zinc	4,369	4,335	162.5	0.888	<0.001	<0.001
Macromineral transfer efficiency^3^ (%; g in milk per 100 g ingested)						
Calcium	24.6	22.9	0.50	0.021	<0.001	<0.001
Magnesium	5.0	4.6	0.09	0.005	<0.001	0.001
Phosphorus	36.1	34.1	1.45	0.049	<0.001	<0.001
Potassium	12.0	10.9	0.39	0.045	<0.001	<0.001
Sodium	30.2	32.2	1.06	0.191	<0.001	<0.001
Trace element transfer efficiency^3^ (%; g in milk per 100 g ingested)						
Iodine	44.6	41.8	2.44	0.434	<0.001	<0.001
Manganese	0.13	0.05	0.004	<0.001	<0.001	<0.001
Molybdenum	4.46	5.25	0.223	0.016	<0.001	<0.001
Zinc	17.0	7.8	0.55	<0.001	<0.001	<0.001

1 n = number of records.

2 Significance was declared at *P* < 0.05.

3 The covariate was not used because wk 0 TMR mineral content was not measured.

**Figure 3:**
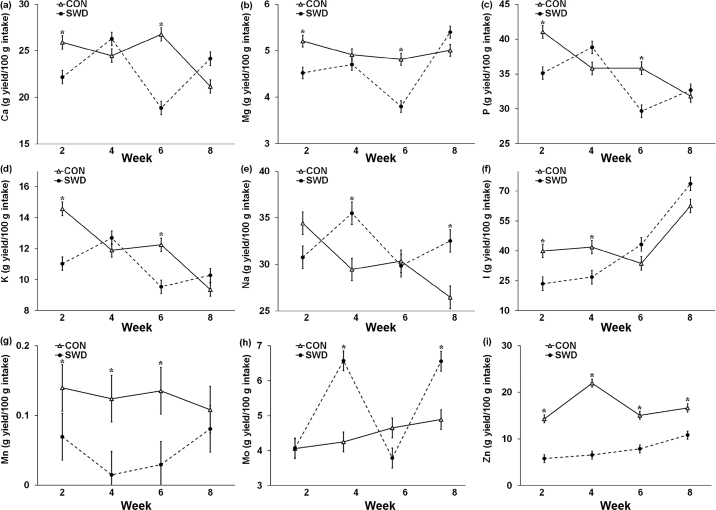
Interaction means ± SE (error bars) for the effects of dietary treatment (control, no seaweed, CON; 330 g/d dried *Ascophyllum nodosum* supplement, SWD) and week (2, 4, 6, and 8) on the transfer efficiencies of Ca (a; g yield/100 g intake), Mg (b; g yield/100 g intake; *P* = 0.001), P (c; g yield/100 g intake; *P* < 0.001), K (d; g yield/100 g intake; *P* < 0.001), Na (e; g yield/100 g intake; *P* < 0.001), I (f; g yield/100 g intake; *P* < 0.001), Mn (g; g yield/100 g intake; *P* < 0.001), Mo (h; g yield/100 g intake; *P* < 0.001), and Zn (i; g yield/100 g intake; *P* < 0.001) from feed to milk. Means for diet treatments within a week denoted with * are significantly different (*P* < 0.05).

There was a significant effect of diet on the feed-to-milk transfer efficiency of Ca (*P* = 0.021), Mg (*P* = 0.005), P (*P* = 0.049), K (*P* = 0.045), Mn (*P* < 0.001), Mo (*P* = 0.016), and Zn (*P* < 0.001; Table 4). For every 100 g of individual mineral intake SWD diet transferred 1.7 g less Ca, 0.4 g less Mg, 2.0 g less P, 1.1 g less K, 0.08 g less Mn, and 9.2 g less Zn, but 0.79 g more Mo, compared with the CON diet. There was a significant effect (*P* ≤ 0.001) for the diet × week interaction for all measured mineral transfer efficiencies (Figure 3). Transfer efficiencies for Ca, Mg, P, and K were lower in SWD cows than in CON cows in wk 2 and 6 but the differences in wk 4 and 8 were not significant. Transfer efficiencies for Na and Mo were higher in SWD cows than in CON cows in wk 4 and 8 but the differences in wk 2 and 6 were not significant. Transfer efficiencies for Zn in CON milk was higher throughout the experiment, but maximum relative difference has been observed in wk 4. Iodine transfer efficiencies from feed to milk were higher in CON cows than in SWD cows in wk 2 and 4, but there was no difference between milk from the 2 experimental groups in wk 6 and 8. The Mn transfer efficiencies were lower in SWD cows than in CON cows in wk 2, 4, and 6.

### Cow Hematological Parameters

Diet did not influence the concentrations of the assessed blood plasma parameters (Table 5).

**Table 5:** Means, SE, and ANOVA *P*-values for the effect of dietary treatment (control, no seaweed, CON; 330 g/d *Ascophyllum nodosum* supplement, SWD) on cows' hematological parameters at the end of the experiment^1^

Parameter	Dietary treatment		SE	ANOVA *P*-value^2^
	CON (n = 23)	SWD (n = 23)		Diet
White blood cells (10^9^/L)	7.38	7.01	0.258	0.320
Neutrophils (10^9^/L)	2.58	2.18	0.172	0.107
Lymphocytes (10^9^/L)	4.45	4.51	0.225	0.843
Monocytes (10^9^/L)	0.117	0.116	0.0120	0.959
Eosinophils (10^9^/L)	0.240	0.210	0.0380	0.579
Neutrophils (%)	34.8	31.1	2.13	0.232
Lymphocytes (%)	60.6	64.3	2.44	0.294
Monocytes (%)	1.57	1.66	0.146	0.691
Eosinophils (%)	3.03	2.94	0.403	0.879
RBC (10^12^/L)	5.91	5.83	0.124	0.625
Hemoglobin (g/dL)	10.5	10.5	0.13	0.697
Hematocrit (%)	29.1	29.1	0.41	0.958
MCV (fL)	49.6	50.2	0.81	0.583
MCH (pg)	17.9	18.2	0.30	0.441
MCHC (g/dL)	36.0	36.2	0.22	0.488
RDW-CV (%)	21.5	20.8	0.44	0.255
PLT (10^9^/L)	231	240	26.0	0.807
MPV (fL)	6.59	6.62	0.113	0.850

1 n = number of records; RBC = red blood cells; MCV = mean cell volume; MCH = mean corpuscular hemoglobin; MCHC = mean corpuscular hemoglobin concentration; RDW-CV = red blood cell distribution width; PLT = platelet count; MPV = mean platelet volume.

2 Significance was declared at *P* < 0.05.

## DISCUSSION

### Effect of Seaweed Supplementation on Milk Production, Milk Composition, and Efficiency Parameters

In the present study, diet seaweed supplementation reduced milk protein concentration and there was a significant diet × week interaction for DMI (kg/d), protein and fat concentrations, and feed efficiency. The 0.06 g/100 g milk average drop in milk protein concentration when cows were fed SWD was similar to that in Newton et al. (2021) when 50 g/d of a brown seaweed mix (91% *A. nodosum*: 9% *L. digitata*) was fed to dairy cows, resulting in a 0.13 g/100 g reduction in milk protein concentration. This small change may be due to the replacement of corn meal in the control diet with *A. nodosum* in the SWD diets and resulting effects on microbial protein synthesis. Qin et al. (2023) saw no change in protein percent when cows were fed 35.7 g/cow per d on a DM basis of *Saccharina latissima*.

There was a significant week effect seen in the current study for basic milk components, but this was expected as stage of lactation also affects milk composition (Laben, 1963; Heck et al., 2009; Forsbäck et al., 2010). Therefore, the observed differences over time could have also been an effect of the lactation stage. Cows within the trial had an average DIM of 161 d, ranging from 34–315 DIM at the start of the experiment, and milk protein concentration typically increases as lactation progresses and milk yield declines (Waite et al., 1956).

### Effect of Seaweed Supplementation on Milk Mineral Concentrations and Associated Transfer Efficiencies

#### Macromineral Concentrations (Ca, Mg, P, K, and Na)

In the present study, diet seaweed supplementation increased milk Mg and P concentrations. This reflects the numerically higher intakes of these 2 minerals from the diet, as SWD cows were fed +9.8 and +10.0 g/d more Mg and P, respectively, than CON cows. Indeed, transfer efficiencies of Mg and P from feed to milk were lower in SWD cows, reflecting the greater intakes of these minerals. Previous work has shown that higher Mg and P intakes can increase their concentrations in milk (Withers et al., 1999; Gustafson et al., 2007). Withers et al. (1999) showed that as farming practices or systems reduced nutrient loss and increased delivery of P in cows' diets, milk P concentrations increased, and another study indicated that an increase in diet Mg intake leads to increased concentrations within raw milk (Zwierzchowski and Ametaj, 2019).

Additionally, seaweed supplementation had a significant negative effect on the transfer efficiencies of macrominerals Ca and K. However, these decreases in transfer efficiencies did not reflect in lower concentrations of Ca or K in milk, again due to the higher intakes of Ca and K from SWD diets. In the case of K, this would have been further explained by the fact that most ingested K is excreted in urine, rather than milk, a relationship that is not true for Ca (Williams et al., 1990; Martín-Tereso and Verstegen, 2011). By nature of the physiological process of excretion from ingested minerals into milk, the proportion of diversion toward urine and feces over milk increases as intake increases (López-Alonso, 2012). Therefore, a slight increase in the macrominerals intake for the SWD group may have led to reduced transfer efficiency, with SWD cows experiencing a drop of 1.7%, 0.4%, 2.0%, and 1.1% for Ca, Mg, P, and K, respectively, similar to Qin et al. (2023), which saw significantly reduced transfer efficiencies by 1.1% and 1.2% for Ca and Na, respectively, when comparing a control diet with a diet containing 35.7 g/cow per d on a DM basis of *S. latissima*.

An effect of diet × week interaction was found to be significant for all measured macrominerals. However, there were no consistent patterns and the relative differences between the experimental groups were 6% to 20% variation of all measured weeks within each group. The between-week variation tended to be higher in SWD milk than in CON milk, reflecting the higher intakes, while there were contradictory relationships between CON and SWD milk macromineral concentrations (e.g., Ca being higher in CON in wk 4 but lower in wk 6 when compared with SWD milk).

#### Trace Element Concentrations (I, Mn, Mo, and Zn)

In the present study, seaweed supplementation had a significant effect on milk I concentrations. Antaya et al. (2019) also reported a rise in milk I by roughly 4 times that of control when cows were fed 113 g/d of *A. nodosum*. In a separate study (Antaya et al., 2015) milk I concentrations reached 1,370 μg/L when cows were fed 170 g/d of *A. nodosum* meal. Qin et al. (2023) reported increases of from 208 to 695 μg/kg milk I when cows were fed *S. latissima*. Other work has reported even higher I concentrations (2,471 µg/kg) in milk of cows fed 50 g/d of *A. nodosum* and *L. digitata*. Given the lack of goitrogenic compounds within the diet, such as thiocyanate, glucosinolates, or goitrin mainly found in rapeseed or cruciferous vegetables among others, milk I concentrations are positively correlated with diet I concentrations (Flachowsky et al., 2014; Bertinato, 2021). In the present study, analysis of TMR I concentrations in wk 2 to 9 showed 5,625 μg/kg DM for the SWD group and 472 μg/kg DM for the CON group, with I intakes averaging 153 mg/d for the SWD group and 12 mg/d for the CON group. Interestingly, the difference between milk I concentrations between SWD and CON milk was incrementally increasing throughout the period of SWD feeding in the present experiment, indicating that these differences may have increased further if SWD feeding had continued for longer, although the effect beyond wk 9 was not investigated in the present study. Previous work found milk I concentration was maximized within 6 wk when larger amounts of I were fed (12.3 mg/kg DM compared with ∼5.6 mg/kg DM in the present work), which was assumed to be due to the Wolff-Chaikoff effect in which over time excessive I triggers reduced absorption and thus reduced excretion through milk (Newton et al., 2021). The pattern of increasing milk I concentrations within the SWD group without an abrupt reduction in transfer efficiency may indicate that the Wolff-Chaikoff effect has not been triggered physiologically during the sampling period.

Seaweed supplementation reduced transfer efficiency of Mn and Zn, and this can be explained by the fact that larger intakes per day would lead to lower transfer efficiencies, as previously shown by Gustafson et al. (2007). For Mn and Zn, SWD added +1,360 and +989 mg/d, respectively, compared with the CON diet. In the present study differences in calculated transfer efficiency were not associated with changes in milk concentrations of Mn, Zn, and Mo. Interestingly, I transfer efficiencies were increased across the experiment for the SWD group. This is a phenomenon also experienced to some degree in Newton et al. (2021), wherein the introduction of a diet containing high amounts of I (via a *A. nodosum* and *L. digitata*) initially reduced transfer efficiency of I, but then stabilized potentially because the amounts were not high enough to trigger the Wolf-Chaikoff effect that would divert I from the mammary gland to the kidneys.

### Effect of Seaweed Supplementation on Cow Health Indicators

The effect of seaweed supplementation did not affect any of the assessed hematological parameters. The parameters assessed in the present study are related to clinical anemia, renal insufficiency, myeloproliferative disorders, and hyperthyroidism among other conditions (Roland et al., 2014). The present work provides evidence that *A. nodosum* can be offered up to 330 g/d in dairy cows without a negative impact on these parameters. The SWD diet was specifically designed to feed I below the EFSA's upper limit of 5 mg I/kg complete feed (EFSA, 2013), but SWD cows consumed on average ∼6.1 mg I/kg DM as a result of (1) discrepancies between book values (used to develop the experimental diets) and the actual I concentrations in the feed ingredients, and (2) the fact that the predicted forage intake was lower than the actual forage intake, thus increasing the relative contribution (g/kg DM) of SWD-containing concentrate in the total diet. Although the diet I concentration in the present study was lower than in previous work (Newton et al., 2021; Qin et al., 2023), these results emphasize the need to measure feedstuff I content when SWD is fed, to ensure that over-supplementation of I does not occur.

### Nutritional Implications of Milk from Seaweed-Fed Cows for Consumers

The ANIBES report (anthropometric data, macronutrient and micronutrient intake, practice of physical activity, socioeconomic data, and lifestyles in Spain) was used in calculations requiring the average milk consumption rates of males and females in Spain by age group (Partearroyo et al., 2019); the EFSA's DRV Finder: Dietary Reference Values for the EU presents an adequate intake of I as 105, 125, 150, and 200 μg/d for individuals aged 9–12, 13–17, and 18+ yr, and pregnant or nursing women, respectively (EFSA, 2019). Therefore, based on the recorded liquid milk intakes from the ANIBES report and the milk I concentrations in the present study, CON milk would contribute (expressed as % adequate intake) (1) 40, 32, 18, and 20 in men of age groups 9–12, 13–17, 18–64, and 65–75 yr, respectively, (2) 32, 23, 19, and 21 in women of age groups 9–12, 13–17, 18–64, and 65–75 yr, respectively, and (3) 14% in pregnant or nursing women. On the other hand, average consumption of milk from the SWD group would substantially exceed the adequate intake of I in all cases (expressed as % adequate intake) (1) 453, 364, 201, and 222 in males of age groups 9–12, 13–17, 18–64, and 65–75 yr, respectively, (2) 367, 258, 215, and 241 in females of age groups 9–12, 13–17, 18–64, and 65–75 yr, respectively, and (3) 161 in pregnant or nursing women. Previous work in Spain (Donnay and Vila, 2012) reported that milk and dairy products are a major contributor to the reduction of the previously observed I deficiency, and the population of Spain generally maintains optimum I nutrition. However, previous reports have indicated low urinary I levels in certain demographics in Spain (37% of children surveyed had below 100 µg/L; Ansótegui and Knörr, 2012). The SWD milk produced in the present study would provide a substantial amount of I, and if such levels also appear within retail products this could provide a strategy to enhance public I sufficiency with special considerations for demographics reported as deficient (children; Ansótegui and Knörr, 2012) or having higher requirement for daily I intakes such as pregnant or nursing women (de Escobar et al., 2007; EFSA, 2019). Iodine status is crucial for pregnant women but also for women who may become pregnant, as it influences fetal development from conception, thus making it important to keep an adequate I status throughout as pregnant women may not be aware of their pregnancy status, and make essential dietary changes, for weeks after conception (Branum and Ahrens, 2017). Milk with increased I concentrations could be a gateway toward increased I supply, particularly within these vulnerable populations. However, several studies have identified the potential risk when feeding SWD to dairy cows of producing milk with such high I concentrations that certain consumer demographics (especially young children) would reach their upper tolerable limit (**UL**) under typical daily intakes, which may also be the case for adults with higher than typical dairy consumption (Newton et al., 2021, 2022; Qin et al., 2023).

The UL for I set by EFSA is 200, 250, 300, 450, 500, and 600 μg/d for the age groups of 1–3, 4–6, 7–10, 11–14, 15–17, and 18+ yr (including pregnant/lactating women), respectively, with there being no difference of sex. In the present study, based on the I concentrations of SWD milk and the current milk intakes of the Spanish population, the contribution toward UL if all milk consumed was from SWD-fed cows would be 117%, 86%, 52%, and 58% for the age groups 9–12, 13–17, 18–64, and 65–75 yr, respectively. For the same age groups, it would require consumption of 207 mL/d (already met with average consumption), 261 mL/d (+20.6% average consumption), 331 mL/d (+99.6% average consumption), and 331 mL/d (+79.3% average consumption) to reach their UL. This highlights the risk of I overconsumption, especially in children, adolescents, or individuals with high dairy consumption if the I concentrations of the SWD milk from the present study was seen at the retail level. The amounts required to reach the UL are not only realistic, but already higher (for children) or very similar (for adolescents) to those already consumed by these demographics. The risk would be even higher for toddlers (age 1–3 yr), who (at a UL of 200 μg/d; EFSA, 2019) would reach their UL by drinking only 110 mL/d, an amount that most toddlers exceed, or would be recommended to exceed, as part of their daily diet.

Excess I intake causes the Wolff-Chaikoff effect mentioned above, a regulatory process that reduces thyroidal hormone synthesis (generally lasting for 24 h) in vulnerable individuals (e.g., those with autoimmune disease, subacute thyroiditis, or a hemithyroidectomy), and failure of adaptation to this regulatory event can lead to transient or even permanent thyroidal dysfunction (Pramyothin et al., 2011; Leung and Braverman, 2014). Additionally, the consequences of I overconsumption during pregnancy are not well understood, and while targeted I delivery for pregnant women is crucial for preventing fetal neurodevelopmental problems (Zimmermann, 2012), the limited ability of the fetus to cope with excess I may also cause issues such as neonatal airway obstruction due to goiter size or congenital hypothyroidism (Farebrother et al., 2019). However, even at I concentrations at the SWD milk in this study, it would require 331 mL/d for a pregnant woman to reach their UL, and the risk of I overconsumption from milk from SWD-fed cows would be much lower than that for toddlers or adolescents. On the contrary, CON milk would only contribute 10%, 8%, 5%, and 5% of the UL for the age groups 9–12, 13–17, 18–64, and 65–75 yr, respectively, and would require consumption of 2,260, 2,976, 3,759, and 3,759 mL/d to reach their UL. These amounts appear to be very high, and it appears that CON milk can be a good source of I in Spanish diets, without posing any risks around increased I intake.

Milk I concentrations were highly variable between diets and between weeks within the same diet. Milk from the CON group averaged 166 μg/kg milk, which is slightly lower for previous studies in Spain that showed concentrations typically higher than 205 μg/kg (Donnay and Vila, 2012). Therefore, when considering the implications of the contribution of milk to I intakes in Spanish population in the current study (which uses CON milk for the control milk I concentration), these may be slightly lower than that typically found. Other studies have also reported that there might be a large between-country variation (34–550 μg/kg in data from 20 industrialized countries; van der Reijden et al., 2017), while within-country geographical and seasonal variation has also been high in previous work in the United Kingdom (<0.01–1,604 μg/kg; Coneyworth et al., 2020) and the United States (∼129–687 μg/kg; Roseland et al., 2020). This indicates the need for country-specific, or even region-specific, research on the contribution of milk and dairy products on I supply to the population. In addition, it should be noted that estimates in the present study have assumed that all milk consumed came from cows fed SWD. In practice, this is unlikely to happen and milk from farms using SWD would likely have their milk bulked at the dairy plant with milk from other farms not feeding SWD, which would reduce I concentration of the raw milk that is processed.

Milk Mg concentrations in the current study did not differ when SWD was fed to an extent that would have a meaningful impact on human nutrition or health. Based on consumer milk intakes, and the most extreme possible difference in Mg concentrations in CON versus SWD milk (wk 2, 91 mg/kg vs. wk 8, 117 mg/kg, respectively), drinking CON or SWD milk would provide 6.1% or 7.8% of adequate intake, respectively, averaged across demographics. Similarly, milk P concentrations did not differ to an extent that would have a meaningful effect on human nutrition or health. Based on consumer milk intakes, and the most extreme possible difference in P concentrations in CON versus SWD milk (wk 8, 1,044 mg/kg vs. wk 4, 1,198 mg/kg, respectively), drinking CON or SWD milk would provide for 35.7% or 41.0% of adequate intake, respectively, across demographics.

## CONCLUSIONS

Seaweed (*Ascophyllum nodosum*) inclusion in dairy cow diets did not affect productivity and feed efficiency, or measured hematological parameters, but increased the milk concentrations of Mg, P, and I. These effects may be explained by the higher intakes when corn meal was substituted with seaweed in dairy cows' diets, as transfer efficiencies from feed to milk were similar (in case of I) or lower (in case of Mg and P). Milk concentrations of Ca, K, Mn, Zn, and Mo were not affected by feeding seaweed in the present study. Based on reported Spanish population milk intakes, the contribution of milk toward I supply would be increased substantially when seaweed is fed to dairy cows, which can be advantageous for consumers with higher I requirements or demographics known to be deficient (children, pregnant and nursing women, or women of childbearing age). However, care should be taken when feeding seaweed to dairy cows to avoid excessive I intakes, as this would result to milk I concentrations that could lead to I overconsumption by children.
